# Metagenome-wide association study of gut microbiome revealed potential microbial marker set for diagnosis of pediatric myasthenia gravis

**DOI:** 10.1186/s12916-021-02034-0

**Published:** 2021-07-08

**Authors:** Peng Liu, Yiqi Jiang, Shanshan Gu, Yinping Xue, Hongxia Yang, Yongzhao Li, Yaxuan Wang, Congya Yan, Pei Jia, Xiaoting Lin, Guoyan Qi

**Affiliations:** 1 Center of Treatment of Myasthenia Gravis, People’s Hospital of Shijiazhuang affiliated to Hebei Medical University, Shijiazhuang, China; 2 Hebei Provincial Key Laboratory of Myasthenia Gravis, Shijiazhuang, China; 3 City University of Hong Kong, Hong Kong, China; 4 Hebei Provincial Clinical Research Center for Myasthenia gravis, Shijiazhuang, China

**Keywords:** Myasthenia gravis, Metagenomics, SCFAs, Adenovirus, Microbial marker

## Abstract

**Background:**

Myasthenia gravis (MG) is an acquired immune-mediated disorder of the neuromuscular junction that causes fluctuating skeletal muscle weakness and fatigue. Pediatric MG and adult MG have many different characteristics, and current MG diagnostic methods for children are not quite fit. Previous studies indicate that alterations in the gut microbiota may be associated with adult MG. However, it has not been determined whether the gut microbiota are altered in pediatric MG patients.

**Methods:**

Our study recruited 53 pediatric MG patients and 46 age- and gender-matched healthy controls (HC). We sequenced the fecal samples of recruited individuals using whole-genome shotgun sequencing and analyzed the data with in-house bioinformatics pipeline.

**Results:**

We built an MG disease classifier based on the abundance of five species, *Fusobacterium mortiferum*, *Prevotella stercorea*, *Prevotella copri*, *Megamonas funiformis*, and *Megamonas hypermegale*. The classifier obtained 94% area under the curve (AUC) in cross-validation and 84% AUC in the independent validation cohort. Gut microbiome analysis revealed the presence of human adenovirus F/D in 10 MG patients. Significantly different pathways and gene families between MG patients and HC belonged to *P. copri, Clostridium bartlettii*, and *Bacteroides massiliensis*. Based on functional annotation, we found that the gut microbiome affects the production of short-chain fatty acids (SCFAs), and we confirmed the decrease in SCFA levels in pediatric MG patients via serum tests.

**Conclusions:**

The study indicated that altered fecal microbiota might play vital roles in pediatric MG’s pathogenesis by reducing SCFAs. The microbial markers might serve as novel diagnostic methods for pediatric MG.

**Supplementary Information:**

The online version contains supplementary material available at 10.1186/s12916-021-02034-0.

## Background

Myasthenia gravis (MG) is a typical autoimmune disease that targets the neuromuscular junction by autoantibodies and is clinically manifested as skeletal muscle fatigue [1]. The prevalence of MG is 150–250 cases per million people [2]. There are two types, ocular MG and generalized MG, depending on whether the myasthenic weakness is limited to the ocular muscles. Approximately 80% of generalized MG cases and only 50% of patients with ocular MG have detectable antibodies against the acetylcholine receptor (AChR) [3, 4]. Pediatric MG accounts for about 10–15% of all MG cases in Europe and North America [5], but is relatively common in China, where up to 50% of patients have disease onset under 15 years [6]. There are many different characteristics between pediatric and adult MG, including symptoms, clinical severity, and antibody titer [7]. Approximately 80% of pediatric MG is ocular MG in China [6]. The diagnosis of pediatric MG can be challenging, because most children do not respond well to MG’s conventional diagnostic methods, such as fatigue test, repetitive nerve stimulation, and neostigmine test.

The gastrointestinal tract is a complex ecosystem containing many resident microorganisms [8], which is a symbiotic relationship with the host, and affects human nutrition, metabolism, and immune function [9–11]. The gut microbiome’s impact on human health is becoming increasingly apparent; an imbalance in its composition may contribute to various diseases, including obesity, inflammatory bowel disease, and immune diseases [12]. Sequencing methods of microbiome initially focused on amplicon sequencing of the 16S ribosomal RNA (rRNA) gene. However, nowadays, it is recommended to perform a genome-wide association study (MWAS) based on the microbiome’s whole-genome shotgun sequencing. This method can detect the microbiome’s genome composition at the species level of bacteria, archaea, fungi, and even viruses and analyze its functional biological characteristics [13]. Recent studies have reported that the gut microbiota in MG patients is altered compared to healthy people [14, 15], and differential gut microbiota and fecal metabolites are related to the clinical subtypes of MG [16]. However, the studies are based on 16S research focusing on adult patients. Therefore, we present our work for pediatric MG patients with whole-genome shotgun sequencing data. Key metabolites produced by gut microbiota are short-chain fatty acids (SCFAs) [17], in which content in fecal is significantly lower in the adult MG group [14]. We will explore the relationship between pediatric MG’s gut microbiome and the SCFAs.

In this study, we identified potential microbial marker species that could be used for MG identification by whole-genome shotgun sequencing analysis of fecal samples of pediatric MG patients and healthy controls (HC). Based on functional annotation, we found that the gut microbiome affects the production of short-chain fatty acids (SCFAs). We confirmed this result by determining SCFA levels in the serum of the participants. Our study elucidates the underlying roles and mechanisms of gut microbiota in pediatric MG pathogenesis. The novel microbiota-targeted markers will significantly help for the diagnosis of pediatric MG.

## Methods

### Inclusion criteria

We recruited a discovery cohort of 53 pediatric MG patients and 46 HC that were matched for age and gender; this study was conducted at the Center of Treatment of Myasthenia Gravis Hebei Province, People’s Hospital of Shijiazhuang. Clinical, grading of all patients was class I (any ocular muscle weakness; may have weakness of eye closure; all other muscle strength is normal.), based on the Myasthenia Gravis Foundation of America (MGFA) clinical classification [18]. The diagnosis of pediatric MG was based on the clinical presentation of the patients and was confirmed with at least one diagnostic test, including positive antibodies (AChRAb), electromyography, or fatigue test and response to a therapeutic trial (Additional file 4: Table s-1). We further recruited 19 pediatric MG patients as the validation cohort based on the same criteria. Participants were excluded if they had one of these conditions: (1) age < 2 years and 10 months, or no age information; (2) antibiotic usage (except β-lactam) within 3 months of the study; (3) received any MG-related treatments (e.g., pyridostigmine, glucocorticoid, human immunoglobulin, or Chinese herbal medicine); (4) using of any drugs or unknown status; (5) with a family history or onset after birth; and (6) complicated with other diseases. The study was reviewed and approved by the ethics committee of the People’s Hospital of Shijiazhuang (NO. 66). Informed consent was obtained from all recruited patients’ parents and healthy individuals’ parents.

### Fecal sample collection

Approximately 2 g of a fresh fecal sample was collected in a Fecal collection tube (OMR-200 DNA Genotek) and stored at room temperature until DNA extraction (21–28 days). The instruction of OMR-200 DNA Genotek showed the samples could store at room temperature for 60 days (https://www.dnagenotek.com/us/products/collection-microbiome/omnigene-gut/OMR-200.html). Samples were collected before patients were treated.

### DNA extraction and sequencing

The DNA extraction and library construction are followed the instruction of TruSeq DNA Nano Reference Guide (1000000040135) (https://sapac.support.illumina.com/downloads/truseq-dna-nano-reference-guide-1000000040135.html) and Hiseq 2500 System Guide (15035786) (https://support.illumina.com/downloads/hiseq_2500_user_guide_15035786.html) from Illumina. Sterile plastic cupDNA concentration was assessed using a fluorometer reader (Qubit Fluorometer, Invitrogen). DNA sample integrity and purity were confirmed with agarose gel electrophoresis (concentration of agarose gel: 1%, voltage: 150 V, electrophoresis time: 40 min). One microgram genomic DNA from each sample was randomly fragmented by Covaris (LE220), and sequences of an average size of 200–400 bp were selected by magnetic beads (Ampure XP). The fragments were end repaired and 3′-adenylated, and adaptors were ligated to their ends. This process was performed to amplify fragments with the adaptors from the previous step. PCR products were purified using magnetic beads. The double-stranded PCR products were heat denatured and circularized by the splint oligo sequence. The single-strand circular DNA (ssCir DNA) was formatted as the final library. The library was amplified with the phi29 DNA polymerase to make DNA nanoballs (DNBs), which were loaded into the patterned nanoarray. Paired-end 100/150 bp reads were generated via the combinatorial Probe-Anchor Synthesis (cPAS). After library construction, the samples were sequenced on the Illumina HiSeq 2500 platform.

### Quality control

We performed quality control of the sequencing data according to the following steps before analyses: (1) filtered low-quality reads and (2) removed contamination of the human genome sequence. We used fastp (Version: 0.21.1) [19] with its default parameters to screen out low-quality reads and sequence adapter sequences, aligned the reads to the human genome (hg38) with bowtie2 (Version: 2.3.5) [20], and used samtools (Version: 1.9) to screen out paired reads that did not align to the human genome. Clean data were used in subsequent analyses.

### Taxonomy annotation and functional annotation

We used metaphlan2 (Version: 2.1.0) to map high-quality reads to the mpa_v20 marker gene database according to the metagenomics data analysis pipeline of The Huttenhower Lab; this enabled us to obtain microbial taxonomy abundance profiles at different taxonomic levels for each sample. We used the utils of metaphlan2 merge_metaphlan_tables.py to combine the results of all samples and utilized an in-house script to generate a combined profiling table for different taxonomic levels. Humann2 (Version: 2.0) was used to map high-quality reads to uniref90 and chocophlan (humann2 recommended databases) for profiling of gene family abundance and pathway abundance. We subsequently used the humann2 utils: humann2_join_table(s), humann2_renorm_table, and humann2_split_stratified_table to merge the abundance of all samples, normalize the abundance, and to stratify profiles with taxonomy annotations, respectively.

### Statistical analysis

After obtaining the species and functional abundance profiles, we used the Wilcox.test two.sided function in R (Version: 3.5.3) to assess differences in bacterial abundance between the two groups. *P* values in the results were corrected according to the Benjamini–Hochberg (BH) method to obtain q values (false discovery rate, FDR), which were used for identifying significantly different species and pathways between the pediatric MG patients and HC.

For the calculation of the Shannon index, we used in-house scripts to calculate the α-diversity of each sample using the taxonomy abundance data. Using the same input data, the vegdist method of the Vegan package (Version: 2.5-5) in R (with the parameter “method = dist_method”) was used to calculate β-diversity.

To explore relationships among samples, we performed principal component analysis (PCA) and principal coordinate analysis (PCoA) with the taxonomy abundance data using the Ade4 package (Version: 1.7-15) of R. We use the corr.test of the R package with “method = spearman, use = pairwise, adjust = BH” to calculate the Spearman’s rank correlation between the clinical phenotypes and significantly different microbial species and functions.

### Random forest model

Significantly different microorganisms between MG patients and HC at all taxonomy levels were assigned as candidate microbial markers. Using the relative abundance of the candidate markers at each taxonomy level, we used a random forest model to build classifiers to distinguish between HC and MG patients with RandomForest package (Version: 4.6-14) in R. The samples we used in this step were labeled with the two-value disease situation. We applied the package in classification with default parameters, like the number of trees grow in the process is 500. Subsequently, a fivefold cross-validation method was used to evaluate the performance of the predictive model. We calculated the minimum error in the average cross-validation error curve and the average curve and used the standard deviation of this point as the cut-off point of the filter prediction model. The set of candidate markers, which contained the minimum number of candidate markers in all groups and had an error below the critical value, was selected as a marker set to build the diagnostic classifier. The probability of MG was then calculated based on this optimal set; the receiver operating characteristics (ROC) of the discovery and validation cohort were plotted using the pROC package (Version: 1.16.2).

### Quantification and analysis of SCFAs in serum samples

Approximately 2 ml of fresh peripheral blood of MG patients and HC individuals were collected in the early morning before meals. Samples were collected before patients were treated. These samples were centrifuged at 3000 rpm for 10 min after being left at room temperature for 30 min; the serum was collected and stored at − 80 °C until further analysis. The SCFAs quantification protocol was referenced from Sandin et al., Vemuri et al., and Sivaprakasam et al. [21–23].

The standard SCFAs solution was prepared as following: 1 mg/ml SCFAs was diluted with ultrapool water into 1 ml solution with concentrations of 1 μg/ml, 2 μg/ml, 5 μg/ml, 10 μg/ml, 50 μg/ml, 100 μg/ml, and 200 μg/ml, respectively. Take ≥ 50 μL fluid, add 400 μL saturated sodium chloride solution and 50 μL saturated hydrochloric acid sodium chloride solution with 3 mmol, vibrate it to dissolve completely, and use low-temperature ultrasound for 20 min. Then, the standard curve was drawn with the concentration of standard SCFAs (μg/ml) as the horizontal coordinate and the peak area as the vertical coordinate.

To prepare the testing solution, 50 g samples were mixed with 400 μL saturated sodium chloride solution and 50 μL with 3 mmol saturated hydrochloric acid sodium chloride solution. The fully dissolved mixed solution was carried on ice bath ultrasound for 20 min. After the ultrasound, 400 μL ice ether was added and the mixed solution was shaken for 10 min to fully extract. The solution was then centrifuged for 10 min at 12000 rpm at 4 °C. Collect the supernatant and add 50 mg anhydrous sodium sulfate to oscillate for 3 min and centrifuge for 5 min at 4500 rpm at 4 °C. The supernatant was used in the followed GC-MS analyses.

The analytical instrument used in testings was Thermo Trace1300-Thermo TSQ9000 GC/MS. SIM mode was used for data acquisition, and Tracefinder (Thermo Fisher Scientific, Waltham, MA, USA) was used for data processing.

The column used in gaseous phase was Agilent HP-FFAP (25 m × 0.50 mm × 0.32 μm), the temperature was processed as follows: 100 °C for 1 min and 5 °C/min until 160 °C for 2 min. In mass spectrometry, the ion source temperature was 280 °C. The mass spectrometer was set to scan mode from m/z 35-200 and monitoring in SIM Ion m/z 43,45,60 for acetic acid, m/z 45,57,74 for propanoic acid, m/z 43,73,88 for butyric acid, m/z 41,60,73,88 for isobutyric acid, m/z 43,60,87 for N-Valeric acid, m/z 41,60,73,87 for isovaleric, and m/z 60,73,87 for caproic acid. The Pearson correlation between SCFAs and the abundance of species and the corresponding *p* value were calculated using the Hmisc package of R with default parameters.

## Results

### Human adenovirus identified in the gut microbiome of pediatric MG patients

The discovery cohort comprised 53 pediatric MG patients and 46 age- and gender-matched HC. The clinical information of all patients is given in Additional file 4: Table s-2. In the discovery cohort, the patients and healthy individuals showed no significant difference in clinical indices; there were significantly more female individuals than males in the cohort (Table 1, Wilcoxon test, BH-adjusted *p* < 0.05).

**Table 1:** Basic demographic characteristics of MG patients (*n* = 53) and healthy controls (*n* = 46) in the discovery cohort

Characteristic	MG (*n* = 53)	HC (*n* = 46)	Missing value	Adjusted *p* value
Age	6.64 ± 3.79	7.51 ± 3.79	0	0.16029
Birth mode (%, VD)	29 (54.7%)	33 (71.7%)	1	0.12464
Gender (%, M)	16 (30.2%)	25 (54.3%)	0	0.04688

The possible values of birth mode are vaginal delivery (VD) or cesarean section. Gender could be male (M) or female. The symbol % represent percent

We initially focused on the metagenomic diversity of the MG patients and HC. After quality control filtering of the sequences (Additional file 4: Table s-3), we aligned high-quality reads to the mpa v20 maker gene database of MetaPhlAn2 [24] and obtained the species abundance data for the samples. We used this species abundance profiling data to conduct diversity analysis and detected no differences between patients and HC in alpha diversity (based on Shannon index results at different taxonomic levels) or beta diversity (Additional file 1: Figure s-1). Exploratory PCA and PCoA analyses using the profiling data of different taxonomic levels, from phylum to species, suggested no obvious separation or clustering (Additional file 2: Figure s-2).

**Fig. 1:**
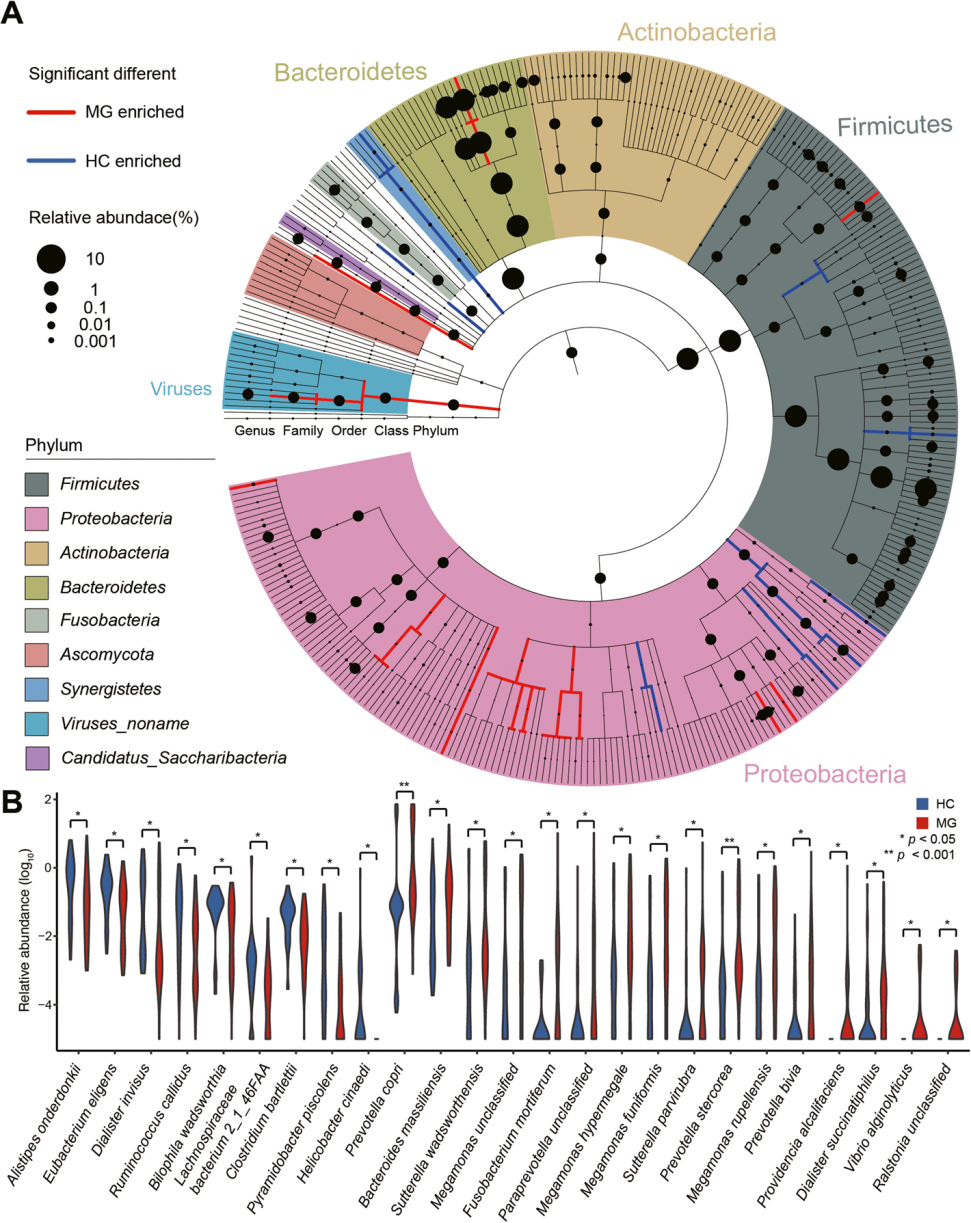
Gut microbiome alterations in pediatric myasthenia gravis patients. **A** Phylogenetic tree. From the inner layer to the outer layer: phylum, class, order, family, and genus. The size of node represents relative abundance. Clades with relative abundance significantly (adjusted *p* < 0.05) enriched in the myasthenia gravis (MG) cohort or the healthy controls (HC) are represented with red or blue color, respectively. **B** Violin plot of relative abundance distribution in MG and HC cohorts of MG-associated species. From left to right, the first four species are enriched in the HC group; other species are enriched in the MG group

We next applied differential tests for abundance profiling of MG and HC groups across six taxonomic levels. At the phylum level, *Deinococcus–Thermus* and *Synergistetes* were enriched in the HC group, while *Cyanobacteria* and *Viruses_noname* were enriched in the MG group (Wilcoxon test, BH adjusted *p* < 0.05) (Fig. 1A). At the genera level, six genera were enriched in the MG group and five genera were enriched in the HC group (Additional file 4: Table s-4).

At the species level, 16 species were enriched in the MG group and nine species were enriched in the HC group (Fig. 1B). Notably, *Prevotella copri* (*P. copri*), which was enriched in the MG patients, has been previously reported as an enriched species in the gut microbiomes of rheumatoid arthritis (RA) patients [25].

Besides bacteria, one virus was also enriched in the MG group. We detected human adenovirus in the microbiomes of 10 MG patients (total 53 MG patients). Nine of them were human adenovirus F and one was human adenovirus D. No healthy individuals contained viral sequences in the data. Since the database used in taxonomy annotation is a marker gene database, we aligned the clean data with the reference genome human adenovirus D (NC_010956.1) and human adenovirus F (NC_001454.1) data from NCBI and confirmed that the viral genome was present in the gut microbiome of these 10 MG patients, with over two average alignment depth of adenovirus reference genomes (Additional file 4: Table s-5). We detected no mapping reads in other samples.

### Co-abundance gene groups reveal four species from *Bacteroides* genus altered in MG gut microbiomes

**Fig. 2:**
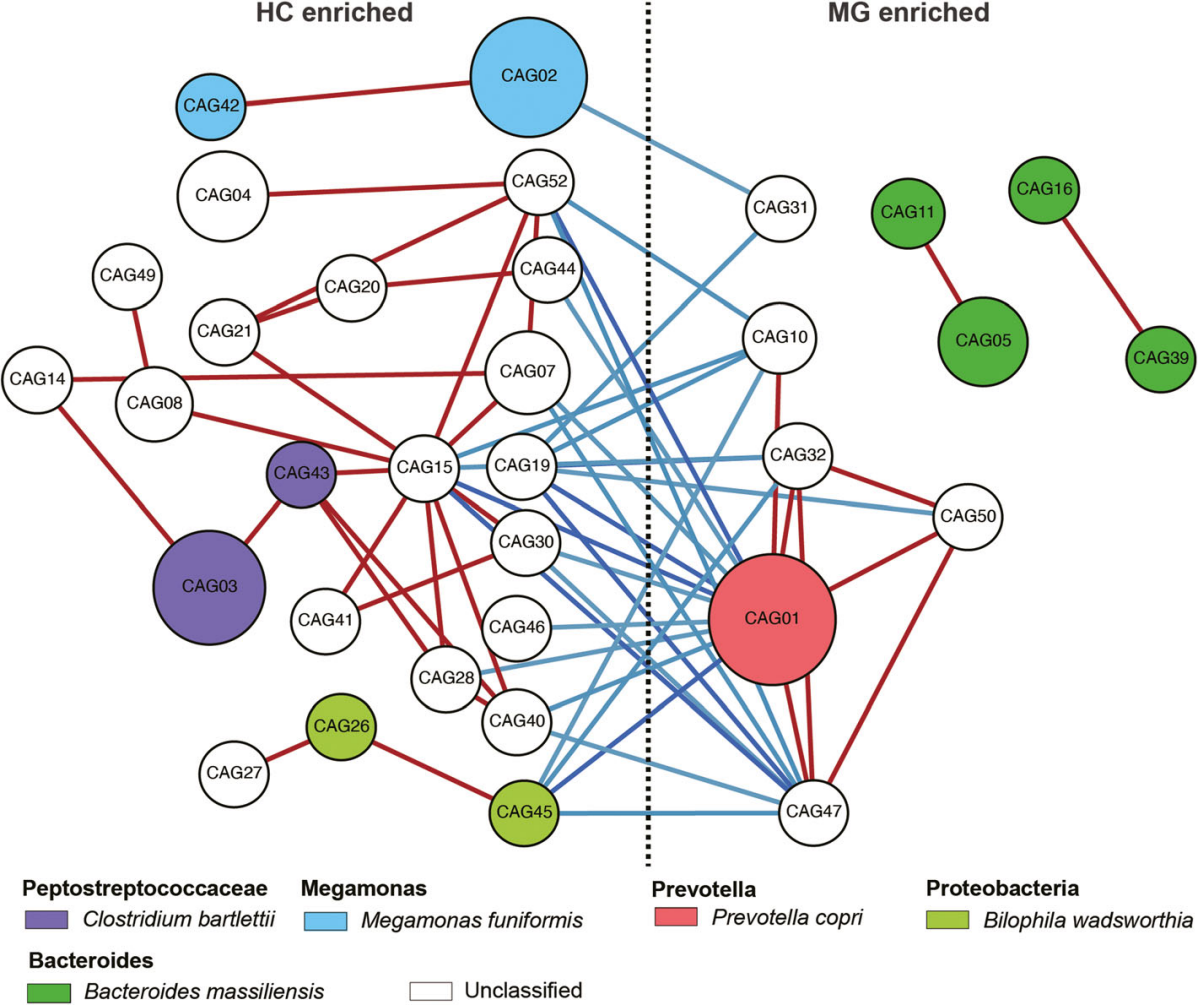
Myasthenia gravis (MG)-associated co-abundance gene groups (CAG) reveal species perform function alter. Correlation network of MG-associated stratified gene families CAG. Size of circles represents gene families contained in this CAG. Colors represent CAG-noted species. Edges in red and blue represent correlation > 0.5 and correlation < − 0.2, respectively. The red scale represents high correlation values, and the blue one represents low correlation values

We detected 12,803 significantly different stratified gene families between the discovery cohort MG patients and the HC group. Using Canopy-based algorithm clustering, 238 co-abundance gene groups (CAGs) were obtained [26]. For each CAG, if 80% of the included gene families were from the same species, the species annotation of this CAG was assigned to this species; otherwise, the annotation was unclassified. We constructed an interaction network for the CAGs based on Pearson correlation values (Fig. 2). The CAGs enriched in the HC group belonged to *C. bartlettii*, *Bilophila wadsworthia*, and *Bacteroides dorei*. The CAGs enriched in the MG group mainly belonged to *P. copri* and *Bacteroides massiliensis*.

### A classifier based on microbial markers species validated in the validation cohort

We next explored whether the gut microbiome profiling data has the potential to distinguish pediatric MG patients from HC, by constructing a disease classifier using a random forest model and the data for the significantly different species (Additional file 3: Figure s-3).

In order to evaluate the accuracy of this classification model, an independent cohort consisting of 19 MG patients without HC samples was used as the validation cohort. To ensure the same proportion of the two types of samples in the training set and the test set, we randomly selected 12 HC samples from the discovery cohort and 19 MG patients in the validation cohort to form the test set, and the remaining 53 MG patients and 34 HC samples were used as the training set.

**Fig. 3:**
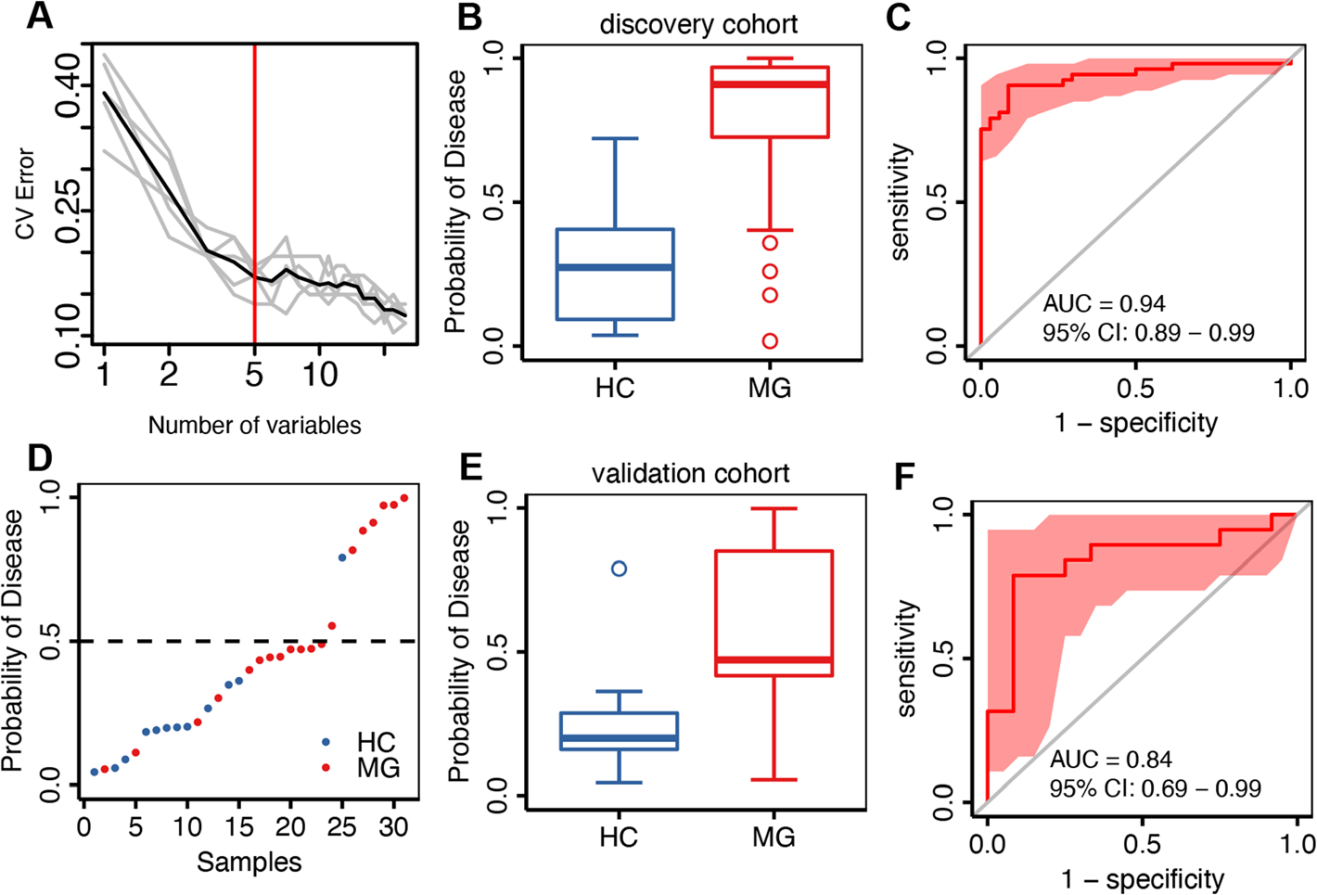
Classification of myasthenia gravis (MG) status by relative abundance of candidate microbial marker species. **A** Distribution from five trials of 10-fold cross-validation error in a random forest classification of MG, with increasing species number used in each model. The model was trained using the relative abundance values for MG-associated species. The gray lines represent the cross-validation error for the five trails. The black curve represents the average of the gray lines. The red line marks the number of species in the optimal marker species set. **B** The probability of MG in the discovery cohort. **C** Receiver operating characteristic (ROC) in the discovery cohort. **D** Classification of the validation samples and their predicted probability of MG. **E** The probability of MG in the validation cohort. **F** ROC for the validation cohort samples

We used this training set to build a disease classifier using the 25 different species obtained in the above-mentioned differential tests; five species were selected as candidate microbial markers, and a classification model was established using a fivefold cross-validation method (Fig. 3A–C). The area under the receiver operating curve (AUC) of the corresponding ROC curve reached 0.94. All five marker species were enriched in the MG group. The bacterial species used for classification include *Fusobacterium mortiferum (F. mortiferum)*, *P. stercorea*, *P. copri*, *M. funiformis*, and *M. hypermegale*. The highest cross-validation average accuracy was found when the markers contained *P. stercorea* and *P. copri*.

The test set achieved an AUC value of 0.84 (Fig. 3D, E). Detailed predicted probability of MG in the discovery cohorts and validation cohorts is listed in Additional file 4: Table s-6. These results suggest that MG-related microbial markers may have the potential for use in non-invasive diagnostic strategies for MG in children.

### AChRAb-positive and AChRAb-negative MG patients reduce SCFA levels through different metabolic pathways

**Fig. 4:**
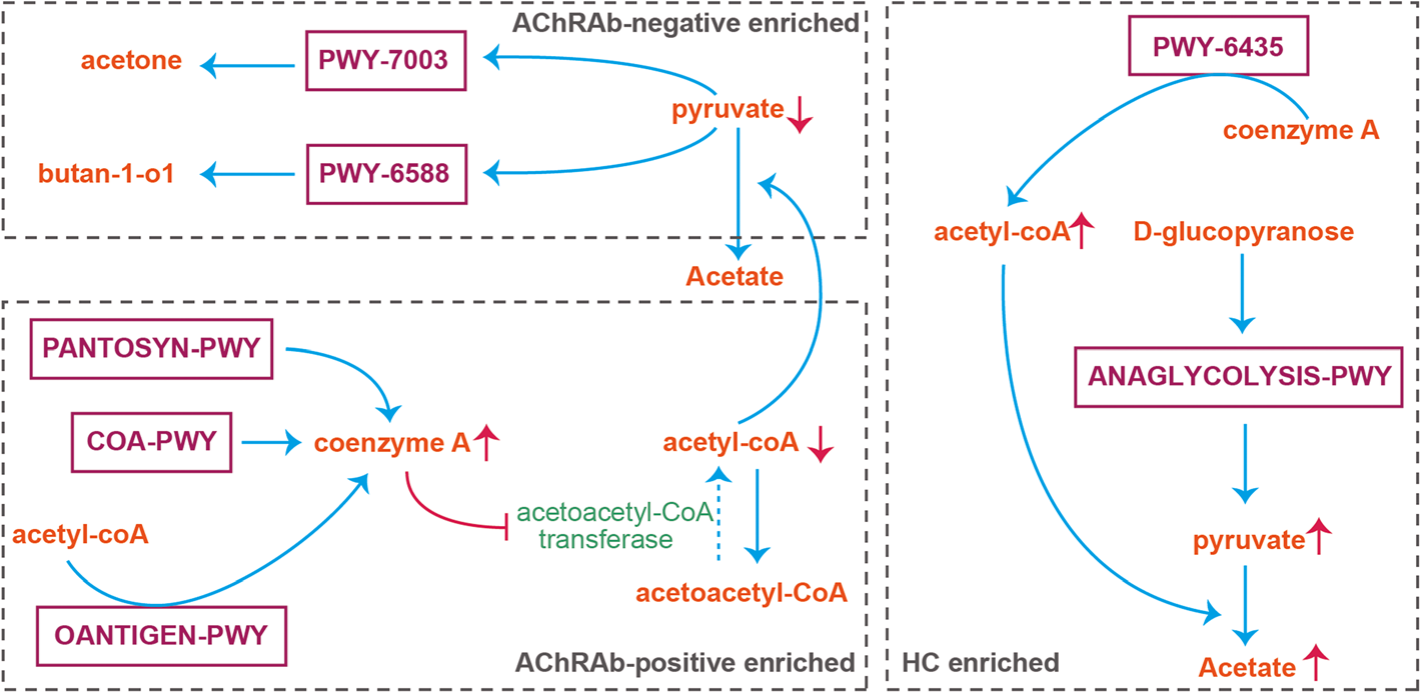
Acetylcholine receptor antibody (AChRAb)-related gut microbiome pathways affect the production of short-chain fatty acids (SCFAs). Orange text denotes the products involved in metabolism. Purple rects represents MetaCyc pathway terms. Blue arrows show the direction of material flow in the network of different pathways. Red arrows show increase/decrease in products. Gray rects split pathways to AChRAb-positive enriched, AChRAb-negative enriched, and HC-enriched

Based on the AChRAb levels in the blood, we divided the MG patients into AChRAb-positive (*n* = 34, AChRAb > 0.5 nmol/L) and AChRAb-negative (*n* = 19) groups (Additional file 4: Table s-1). There were no significant differences in demographic of these two (Additional file 4: Table s-7). We then assessed these two MG groups to explore relationships between AChRAb and the gut microbiomes of the MG patients (Fig. 4).

We first compared the taxonomic abundance data of AChRAb-negative and AChRAb-positive groups with HC at the species level and found that *P. stercorea* was the only significantly different species enriched in the AChRAb-negative patients. However, the abundance of 14 species was significantly different between HC and AChRAb-positive MG patients. The 14 species contained *P. stercorea*, which is a candidate microbial marker species identified in the previous experiment. Moreover, all 14 species were significantly different in abundance in the HC and MG cohorts.

We further compared the abundance of unstratified pathways in HC with the AChRAb-positive and AChRAb-negative MG patients. Of the 16 unstratified pathways with significant differences in abundance between HCs and AChRAb-negative MG patients (Additional file 4: Table s-8), four are related to SCFAs; “ANAGLYCOLYSIS-PWY: glycolysis III (from glucose)” and “PWY-6435: 4-hydroxybenzoate biosynthesis V” were enriched in the HC group, whereas “PWY-7003: glycerol degradation to butanol” and “PWY-6588: pyruvate fermentation to acetone” were enriched in the AChRAb-negative MG patients.

ANAGLYCOLYSIS-PWY: glycolysis III (from glucose) is the main pathway for degrading d-glucopyranose to pyruvate. PWY-6435: 4-hydroxybenzoate biosynthesis V has an intermediate step, with CoA as the enzyme to produce acetyl-CoA, while generation of acetate from pyruvate via acetyl-CoA is the most common pathway of enteric bacteria [27, 28]. The AChRAb-negative enriched pathways are consuming pyruvate. PWY-6588 is pyruvate fermentation pathway, which PWY-7003 will use pyruvate to produce butan-1-ol.

Comparison of HC and AChRAb-positive MG patients revealed 15 significantly different unstratified pathways (Additional file 4: Table s-9): Ten pathways were enriched in AChRAb-positive MG patients and five pathways were enriched in HCs. Four of these pathways were related to SCFAs: COA-PWY: coenzyme A biosynthesis I, OANTIGEN-PWY: O-antigen building blocks biosynthesis (*E. coli*), and PANTOSYN-PWY: pantothenate and coenzyme A biosynthesis I, which were all enriched in AChRAb-positive MG patients, and PWY-6435 was enriched in HCs. Both COA-PWY and PANTOSYN-PWY synthesize CoA, and OANTIGEN-PWY contains a catalysis step by acetyl-CoA and reduces CoA. Due to the competitive relationship, CoA inhibits the enzymatic reactions of the acetoacetyl-CoA transferase, which produces acetate [29, 30], while the HC-enriched pathway PWY-6435 converts CoA to acetyl-CoA.

### Butyric acid and isobutyric acid levels decreased in MG patients

**Fig. 5:**
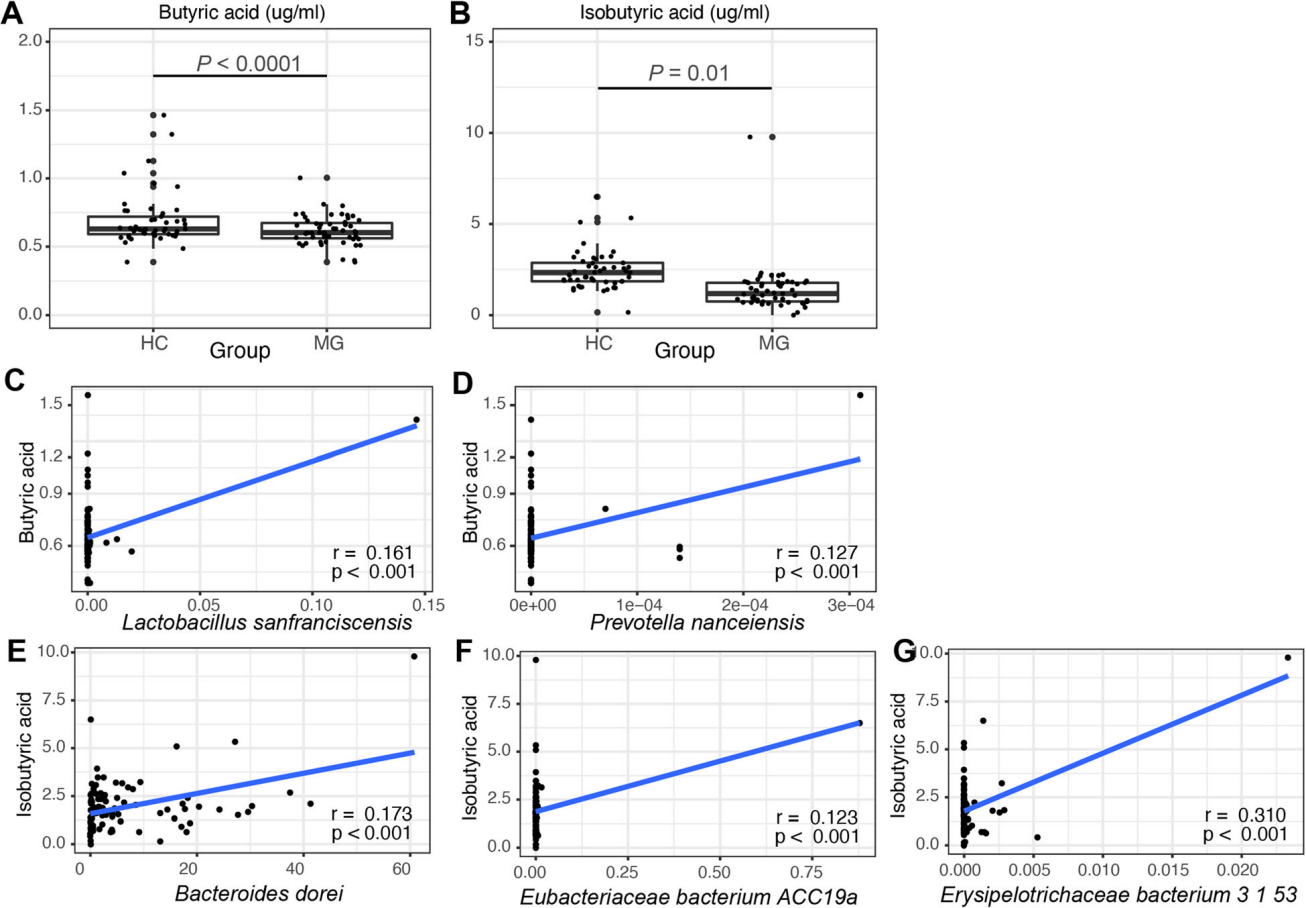
Difference in the level of SCFAs between MG and HC, and the correlations of SCFAs with the enriched species in two groups. Distribution of the levels of butyric acid (**A**) and isobutyric acid (**B**). The *p* value of t test showed significant difference between the two groups. Correlation between the level of butyric acid and *Lactobacillus sanfranciscensis* (**C**) and *Prevotella nanceiensis* (**D**); the level of isobutyric acid and *Bacteroides dorei* (**E**), *Erysipelotrichaceae bacterium 3_1_53* (**F**), and *Eubacteriaceae bacterium ACC19a* (**G**). Pearson correlation (r) and *p* value (p) were denoted. The lines represented the linear regression line

To verify the relationship between MG gut microbiome and SCFAs, we tested the SCFAs quantity in blood samples from the discovery cohort (Additional file 4: Table s-10). This revealed significantly different levels of butyric acid and isobutyric acid between HCs and MG patients (Fig. 5A, B). We further calculated the Pearson correlation between species abundance (species level) and butyric acid and isobutyric acid content. This revealed 13 and 16 species exhibiting significant correlations with blood butyric acid and isobutyric acid content, respectively. The abundance of *Lactobacillus sanfranciscensis* and *Prevotella nanceiensis* is significantly positively correlated with the blood butyric (Fig. 5C, D), and *Bacteroides dorei*, *Erysipelotrichaceae bacterium 3_1_53*, and *Eubacteriaceae bacterium ACC19a* are significantly positively correlated species to blood isobutyric acid (Fig. 5E–G). The abundance of those species is different between HC and MG that we identified above (p before adjusted < 0.05). While *Erysipelotrichaceae bacterium 3_1_53* is the only one enriched in MG, the other four species were enriched in HC.

## Discussion

In this study, we conducted gut microbiome analysis of pediatric MG cases, using whole shotgun genome metagenomics sequencing. We collected samples from a Chinese cohort comprising pediatric MG patients and age-matched HC. We determined the microbial markers that were enriched or depleted in the MG group at different taxonomy levels. Based on this analysis, we identified and tested an MG classifier that can distinguish MG patients and HC based on the abundance of microbial markers. Subsequently, functional analyses implicated several microbiome pathways in the MG cohort. Thus, our study shows how the gut microbiome influences MG, and illustrates excellent microbial markers for diagnosing MG.

Our data indicated that MG patients had no significant differences in alpha diversity indices compared to the HC. This finding is notably inconsistent with a previous study on MG, which is based on 16S research focusing on adult patients [15]. Two plausible explanations could help explain these apparent differences. One is the possibility of systematic error between the 16S rRNA gene sequencing data and metagenomic sequencing data; the second is that our cohort comprised pediatric MG cases, whereas the other examined cohorts in the previous study comprised adult MG cases.

We then conducted a Wilcoxon differential test to detect taxa with significantly different abundance between the MG and HC cohorts. Among the 14 significantly different genera, 10 were enriched in MG patients, and four were absent in the MG group. Based on the identified marker species *F. mortiferum*, *P. stercorea*, *P. copri*, *M. funiformis*, and *M. hypermegale*, we constructed an MG classifier. For cross-validation, the classifier obtained 0.94 AUC. For the independent validation cohort, the classifier achieved 0.84 AUC. This validation demonstrated that gut microbial features could be used to distinguish MG patients from HC.

Recent advances in sequencing and analysis of metagenomic data have facilitated discovering new viruses and improved our ability to catalog viral communities in an unbiased manner [31, 32]. Notably, in addition to bacterial abundance, our data revealed differential viral abundance between the MG and HC cohorts. Specifically, we detected human adenovirus in the gut microbiome of 10 patients; no adenovirus was detected in the HC. The corresponding species-level viruses are human adenovirus D and human adenovirus F. Viral infection has been proposed as a possible important factor in the initial stage of MG. Cavalcante et al. (2010) proposed the link between viral infection (such as Epstein-Barr virus) and thymus pathology [33]. Other viruses, such as cytomegalovirus, human foamy virus, and Nile virus, may also be related to MG [34, 35]. Further research on the intestinal virus could be helpful for revealing the pathogenesis of pediatric MG.

The Metacyc [36] annotation data showed no significantly different unstratified pathways in the comparison between MG and HC cohorts. We divided the MG cases into AChRAb-positive and AChRAb-negative groups according to AChRAb levels, which resulted in the detection of 15 and 16 significantly different unstratified pathways, respectively, with four and three pathways related to the production of SCFAs, respectively. A common aspect of these significantly different unstratified pathways was altered pyruvate and acetate metabolism. In the AChRAb-positive MG patients, acetyl-CoA was reduced, accompanied by an increased amount of coenzyme A. In the AChRAb-negative MG patients, the amount of pyruvate was reduced.

SCFAs are known to mediate various interactions between the gut microbiome and host metabolism [37] .Both prebiotics and probiotics rely on SCFAs to act on the host [38]. Three molecules that comprise more than 95% of the SCFAs in the human intestine are acetate, propionate, and butyrate [39]. In Type 2 diabetes-related studies, the levels of butyrate and propionate in feces were related to the patient’s insulin response and risk of disease [40]. It is also known that gut microbial dysbiosis of inflammatory bowel disease (IBD) patients and a significantly increased number of mucositis cells are related to reducing butyrate-producing microorganisms in the gut [41]. Butyrate can directly affect immune cells in the intestinal mucosa, increase the number and activity of Tregs, inhibit the neutrophils, macrophages, and dendritic cells, and reduce effector T cell activity [42]. To verify the impact of SCFAs, we tested the SCFA levels in the blood and found that butyric acid and isobutyric acid levels were significantly reduced in pediatric MG patients compared to those in the age-matched HCs.

There were some limitations that should be acknowledged. First, the clinical diagnostic potential of the microbial markers identified by us should be confirmed using multicenter independent samples; more MG patients enrolled from different regions might make our results more solid and reasonable. Second, a previous study noted that the levels of SCFAs are not directly equivalent in the blood and the intestinal environment [43]. Intestinal SCFAs testing would provide more evidence for the roles of altered intestinal flora in the pathogenesis of MG. Third, further cell and animal experiments are needed to determine with certainty whether the relationship between the gut microbiome and MG is causal or incidental.

We detected several gut microorganisms whose abundance was significantly related to the level of serum SCFAs, and we should notice the correlation coefficients were at a low level, like around 0.2. In other words, the correlation does exist but not determinants. The difference between serum SCFAs and fecal SCFAs should be a cause to consider. The relationship between SCFAs in serum and fecal is still under research. Serum SCFA is not as intuitive as fecal SCFA, and SCFA metabolism from the gut harbored microorganism. Wolever et al. reported that acetate and propionate had demonstrated a 40% reduction in serum [44]. However, studies in recent years have also shown that serum SCFAs are positively correlated with the level of fecal SCFAs [45] and can reflect the trend of fecal SCFAs to a certain extent [46]. Our results did not strongly link SCFA produced bacteria with serum SCFAs, but still confirmed the reduction of SCFAs in the gut microbiota of MG patients.

## Conclusion

In summary, we identified a group of microbial markers (*Fusobacterium mortiferum*, *Prevotella stercorea*, *Prevotella copri*, *Megamonas funiformis*, and *Megamonas hypermegale*) associated with pediatric MG. Our study demonstrated the potential of pediatric MG diagnosis by our identified microbial markers. Among the microorganisms enriched in MG patients, we found adenovirus for the first time, which is worthy of further investigation. At the same time, the relationship between the SCFAs and MG needs in-depth understanding. Our findings provide a new approach for studying of the pathogenesis of pediatric MG and developing new diagnostic and therapeutic tools for MG.

## Supplementary Information

**Additional file 1: Figure s-1.** Violin plots of alpha diversity and beta diversity distribution in HC and MG. (A-C) Distribution of alpha diversity based on Shannon index of HC and MG at the phylum (A), genus (B), and species (C) levels. (D-F) Distribution of beta diversity based on Bray-Curtis distance in HC, MG, and between the two groups.

**Additional file 2: Figure s-2.** PCoA of relative abundance in different taxonomy level of all participants. The scatter plots show the distribution of samples by PCo1 and PCo2. Red dots and blue triangles represent MG and HC samples, respectively. Boxplots in vertical and horizontal showed distribution different of two groups in PCo1 and PCo2.

**Additional file 3: Figure s-3.** Flowchart.

**Additional file 4: Table s-1.** Acetylcholine receptor antibodies (AChRAb) level and diagnosis of MG patients. **Table s-2.** Basic demographic characteristics of MG patients (*n* = 53) and healthy controls (*n* = 46) in the discovery cohort. **Table s-3.** Data production, quality control, and data utilization ratios. **Table s-4.** MG-associated microorganisms across all taxonomic levels. **Table s-5.** Average alignment depth of adenovirus in all participants. **Table s-6.** The predicted probability of MG in the training set and the test set, according to the 4 microbial markers species selected based on the random forest model. **Table s-7.** Basic demographic characteristics of AChRAb-positive patients (*n* = 34) and AChRAb-negative patients (*n* = 19) in the discovery cohort. **Table s-8.** AChRAb-negative associated unstratified MetaCyc pathways in MG patients. **Table s-9.** AChRAb-positive associated unstratified MetaCyc pathways in MG patients. **Table s-10.** SCFA levels in blood (μg/ml).

## Data Availability

The whole-genome shotgun sequencing data are deposited in the National Center for Biotechnology Information (NCBI) Database (www.ncbi.nlm.nih.gov) with accession number PRJNA688881. Data related to the current article are available from the corresponding author on reasonable request.

## Abbreviations

CAG: Co-abundance gene groups
HC: Healthy controls
MG: Myasthenia gravis
PCA: Principal component analysis
QC: Quality control
RA: Rheumatoid arthritis
ROC: Receiver operating characteristic
SCFA: Short-chain fatty acids
VD: Vaginal delivery
AUC: Area under curve
CoA: Coenzyme A
DNB: DNA nanoball

## References

[1] Gilhus NE, Tzartos S, Evoli A, Palace J, Burns TM, Verschuuren JJGM (2019). Myasthenia gravis. Nat Rev Dis Primers.

[2] Carr AS, Cardwell CR, McCarron PO, McConville J (2010). A systematic review of population based epidemiological studies in Myasthenia Gravis. BMC Neurol.

[3] Gilhus NE, Verschuuren JJ (2015). Myasthenia gravis: subgroup classification and therapeutic strategies. Lancet Neurol.

[4] Kupersmith MJ, Latkany R, Homel P (2003). Development of generalized disease at 2 years in patients with ocular myasthenia gravis. Arch Neurol.

[5] Phillips LH (2004). The epidemiology of myasthenia gravis. Semin Neurol.

[6] Zhang X, Yang M, Xu J, Zhang M, Lang B, Wang W, Vincent A (2007). Clinical and serological study of myasthenia gravis in HuBei Province, China. J Neurol Neurosurg Psychiatry.

[7] Castro D, Derisavifard S, Anderson M, Greene M, Iannaccone S (2013). Juvenile myasthenia gravis: a twenty-year experience. J Clin Neuromuscul Dis.

[8] Huttenhower C, Gevers D, Knight R, Abubucker S, Badger JH, Chinwalla AT (2012). Structure, function and diversity of the healthy human microbiome. Nature..

[9] Macpherson AJ, de Agüero MG, Ganal-Vonarburg SC (2017). How nutrition and the maternal microbiota shape the neonatal immune system. Nat Rev Immunol.

[10] Roy S, Trinchieri G (2017). Microbiota: a key orchestrator of cancer therapy. Nat Rev Cancer.

[11] Zitvogel L, Ma Y, Raoult D, Kroemer G, Gajewski TF (2018). The microbiome in cancer immunotherapy: diagnostic tools and therapeutic strategies. Science.

[12] Tamburini S, Shen N, Wu HC, Clemente JC (2016). The microbiome in early life: implications for health outcomes. Nat Med.

[13] Sharpton TJ (2014). An introduction to the analysis of shotgun metagenomic data. Front Plant Sci.

[14] Qiu D, Xia Z, Jiao X, Deng J, Zhang L, Li J (2018). Altered gut microbiota in myasthenia gravis. Front Microbiol.

[15] Zheng P, Li Y, Wu J, Zhang H, Huang Y, Tan X, Pan J, Duan J, Liang W, Yin B, Deng F, Perry SW, Wong ML, Licinio J, Wei H, Yu G, Xie P (2019). Perturbed microbial ecology in myasthenia gravis: evidence from the gut microbiome and fecal metabolome. Adv Sci.

[16] Tan X, Huang Y, Chai T, Zhao X, Li Y, Wu J (2020). Differential gut microbiota and fecal metabolites related with the clinical subtypes of myasthenia gravis. Front Microbiol.

[17] Cummings JH, Pomare EW, Branch WJ, Naylor CP, Macfarlane GT (1987). Short chain fatty acids in human large intestine, portal, hepatic and venous blood. Gut..

[18] Jaretzki A, Barohn RJ, Ernstoff RM, Kaminski HJ, Keesey JC, Penn AS (2000). Myasthenia gravis: recommendations for clinical research standards. Task Force of the Medical Scientific Advisory Board of the Myasthenia Gravis Foundation of America. Neurology.

[19] Chen S, Zhou Y, Chen Y, Gu J (2018). fastp: an ultra-fast all-in-one FASTQ preprocessor. Bioinformatics.

[20] Langmead B, Salzberg SL (2012). Fast gapped-read alignment with Bowtie 2. Nat Methods.

[21] Sandin A, Bråbäck L, Norin E, Björkstén B (2009). Faecal short chain fatty acid pattern and allergy in early childhood. Acta Paediatr..

[22] Vemuri R, Gundamaraju R, Eri R (2017). Role of lactic acid probiotic bacteria in IBD. Curr Pharm Des..

[23] Sivaprakasam S, Bhutia YD, Ramachandran S, Ganapathy V (2017). Cell-surface and nuclear receptors in the colon as targets for bacterial metabolites and its relevance to colon health. Nutrients.

[24] Truong DT, Franzosa EA, Tickle TL, Scholz M, Weingart G, Pasolli E, Tett A, Huttenhower C, Segata N (2015). MetaPhlAn2 for enhanced metagenomic taxonomic profiling. Nat Methods.

[25] Maeda Y, Kurakawa T, Umemoto E, Motooka D, Ito Y, Gotoh K, Hirota K, Matsushita M, Furuta Y, Narazaki M, Sakaguchi N, Kayama H, Nakamura S, Iida T, Saeki Y, Kumanogoh A, Sakaguchi S, Takeda K (2016). Dysbiosis contributes to arthritis development via activation of autoreactive T cells in the intestine. Arthritis Rheum.

[26] Nielsen HB, Almeida M, Juncker AS, Rasmussen S, Li J, Sunagawa S (2014). Identification and assembly of genomes and genetic elements in complex metagenomic samples without using reference genomes. Nat Biotechnol.

[27] Louis P, Hold GL, Flint HJ (2014). The gut microbiota, bacterial metabolites and colorectal cancer. Nat Rev Microbiol.

[28] Rey FE, Faith JJ, Bain J, Muehlbauer MJ, Stevens RD, Newgard CB, Gordon JI (2010). Dissecting the in vivo metabolic potential of two human gut acetogens. J Biol Chem.

[29] Sramek SJ, Frerman FE, McCormick DJ, Duncombe GR (1977). Substrate-induced conformational changes and half-the-sites reactivity in the Escherichia coli CoA transferase. Arch Biochem Biophys.

[30] Barker H, Jeng I-M, Neff N, Robertson JM, Tam FK, Hosaka S (1978). Butyryl-CoA: acetoacetate CoA-transferase from a lysine-fermenting Clostridium. J Biol Chem.

[31] Monaco CL, Gootenberg DB, Zhao G, Handley SA, Ghebremichael MS, Lim ES, Lankowski A, Baldridge MT, Wilen CB, Flagg M, Norman JM, Keller BC, Luévano JM, Wang D, Boum Y, Martin JN, Hunt PW, Bangsberg DR, Siedner MJ, Kwon DS, Virgin HW (2016). Altered virome and bacterial microbiome in human immunodeficiency virus-associated acquired immunodeficiency syndrome. Cell Host Microbe.

[32] Wommack KE, Bhavsar J, Polson SW, Chen J, Dumas M, Srinivasiah S, Furman M, Jamindar S, Nasko DJ (2012). VIROME: a standard operating procedure for analysis of viral metagenome sequences. Stand Genomic Sci.

[33] Cavalcante P, Barberis M, Cannone M, Baggi F, Antozzi C, Maggi L, Cornelio F, Barbi M, Dido P, Berrih-Aknin S, Mantegazza R, Bernasconi P (2010). Detection of poliovirus-infected macrophages in thymus of patients with myasthenia gravis. Neurology.

[34] Cavalcante P, Cufi P, Mantegazza R, Berrih-Aknin S, Bernasconi P, Le Panse RJAR. Etiology of myasthenia gravis: innate immunity signature in pathological thymus. Autoimmun Rev. 2013;12(9):863–74. 10.1016/j.autrev.2013.03.010. 10.1016/j.autrev.2013.03.010 23535157

[35] Leis AA, Szatmary G, Ross MA, Stokic DS (2014). West nile virus infection and myasthenia gravis. Muscle Nerve.

[36] Krieger CJ, Zhang P, Mueller LA, Wang A, Paley S, Arnaud M (2004). MetaCyc: a multiorganism database of metabolic pathways and enzymes. Nucleic Acids Res.

[37] Sanna S, van Zuydam NR, Mahajan A, Kurilshikov A, Vila AV, Võsa U (2019). Causal relationships among the gut microbiome, short-chain fatty acids and metabolic diseases. Nat Genet.

[38] LeBlanc JG, Chain F, Martín R, Bermúdez-Humarán LG, Courau S, Langella P (2017). Beneficial effects on host energy metabolism of short-chain fatty acids and vitamins produced by commensal and probiotic bacteria. Microb Cell Fact.

[39] Feng W, Ao H, Peng C (2018). Gut microbiota, short-chain fatty acids, and herbal medicines. Front Pharmacol.

[40] Serino M (2019). SCFAs—the thin microbial metabolic line between good and bad. Nat Rev Endocrinol.

[41] van Vliet MJ, Harmsen HJ, de Bont ES, Tissing WJ (2010). The role of intestinal microbiota in the development and severity of chemotherapy-induced mucositis. PLoS Pathog.

[42] Gonçalves P, Araujo JR, Di Santo JP (2018). A cross-talk between microbiota-derived short-chain fatty acids and the host mucosal immune system regulates intestinal homeostasis and inflammatory bowel disease. Inflamm Bowel Dis.

[43] Den Besten G, van Eunen K, Groen AK, Venema K, Reijngoud D-J, Bakker BM (2013). The role of short-chain fatty acids in the interplay between diet, gut microbiota, and host energy metabolism. J Lipid Res.

[44] Wolever TM, Brighenti F, Royall D, Jenkins AL, Jenkins DJ (1989). Effect of rectal infusion of short chain fatty acids in human subjects. Am J Gastroenterol..

[45] Yamamura R, Nakamura K, Kitada N, Aizawa T, Shimizu Y, Nakamura K (2020). Associations of gut microbiota, dietary intake, and serum short-chain fatty acids with fecal short-chain fatty acids. Biosci Microbiota Food Health.

[46] Guo LX, Tong Y, Wang J, Yin G, Huang HS, Zeng L (2020). Determination and comparison of short-chain fatty acids in serum and colon content samples: Alzheimer’s disease rat as a case study. Molecules.

