# Supplementary figures and images for: Metagenome-wide association study of gut microbiome revealed potential microbial marker set for diagnosis of pediatric myasthenia gravis
Source: BMC Med. 2021 Jul 8;19:159. doi: 10.1186/s12916-021-02034-0 (PMC8265136; doi:10.1186/s12916-021-02034-0)

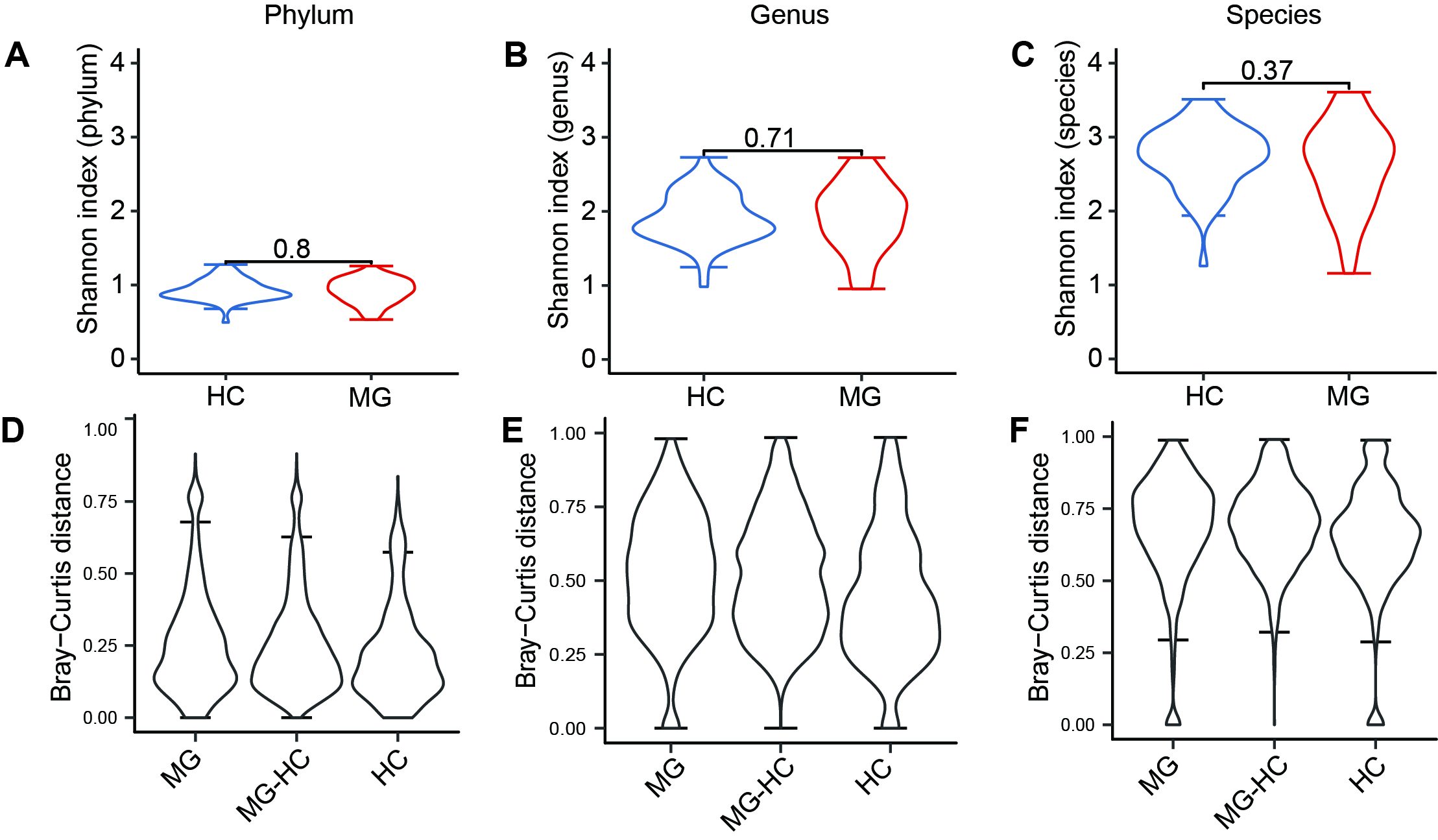

Supplement: Supplementary file 1 — Additional file 1: Figure s-1. Violin plots of alpha diversity and beta diversity distribution in HC and MG. (A-C) Distribution of alpha diversity based on Shannon index of HC and MG at the phylum (A), genus (B), and species (C) levels. (D-F) Distribution of beta diversity based on Bray-Curtis distance in HC, MG, and between the two groups. [file 12916_2021_2034_MOESM1_ESM.tif]

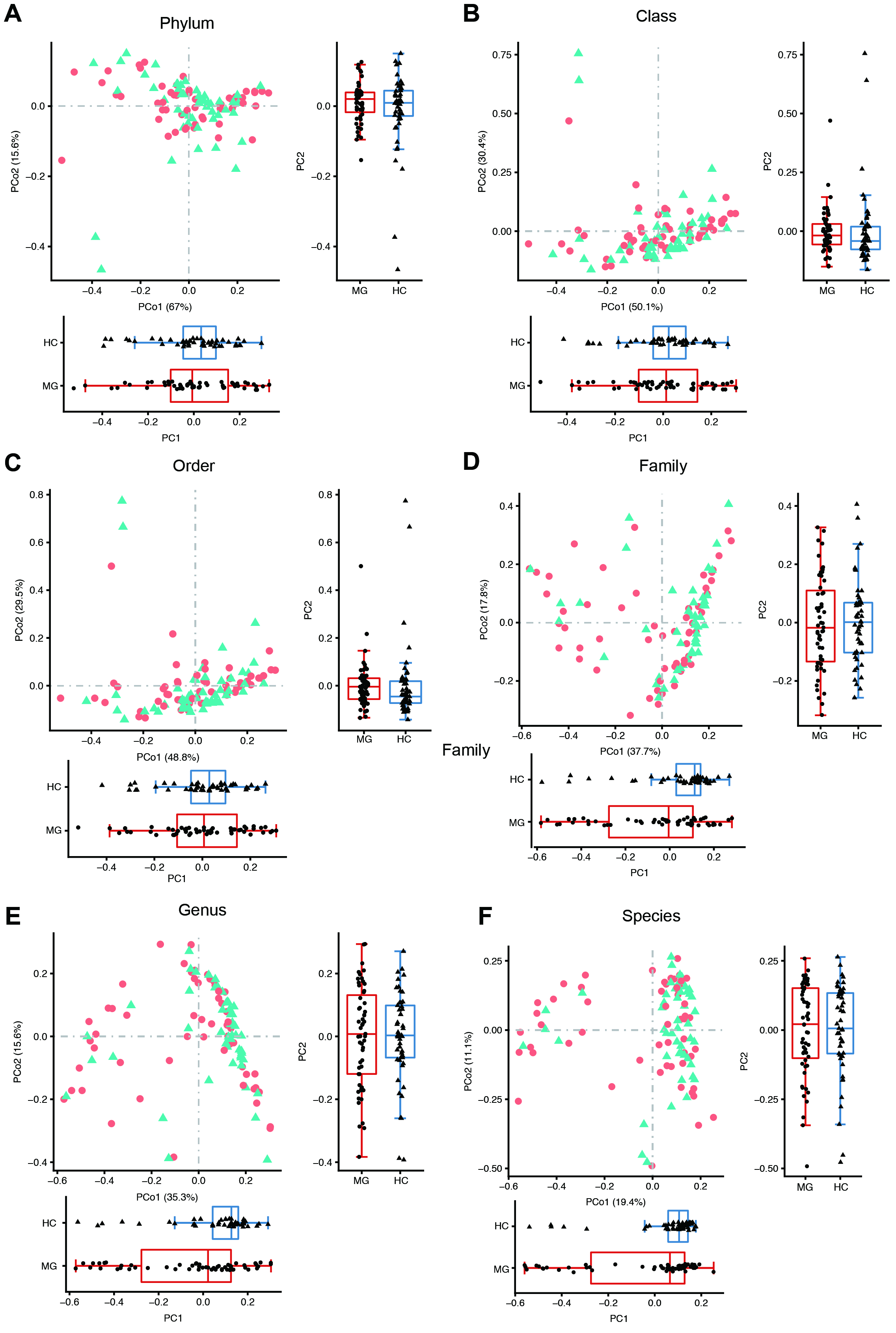

Supplement: Supplementary file 2 — Additional file 2: Figure s-2. PCoA of relative abundance in different taxonomy level of all participants. The scatter plots show the distribution of samples by PCo1 and PCo2. Red dots and blue triangles represent MG and HC samples, respectively. Boxplots in vertical and horizontal showed distribution different of two groups in PCo1 and PCo2. [file 12916_2021_2034_MOESM2_ESM.tif]

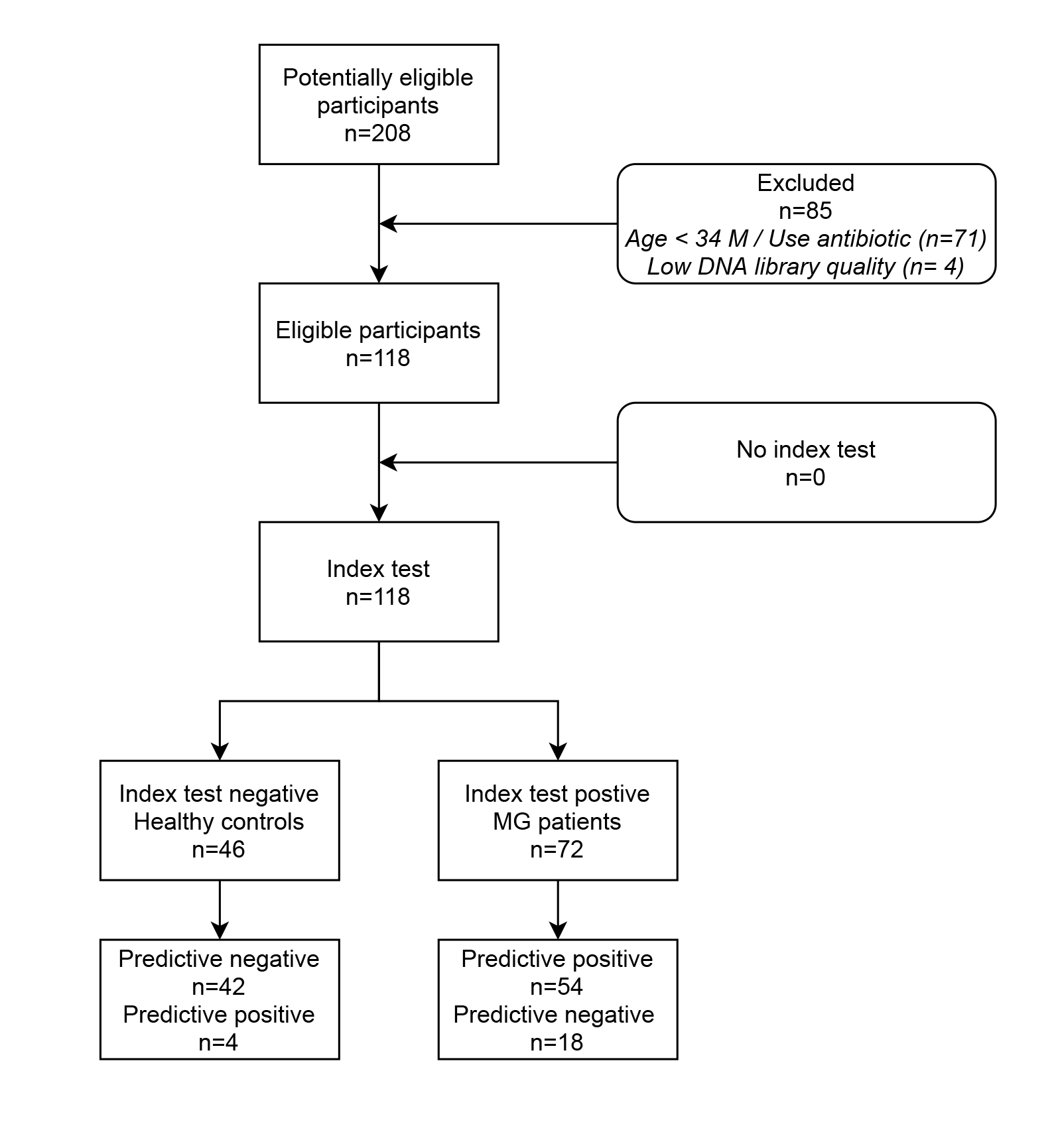

Supplement: Supplementary file 3 — Additional file 3: Figure s-3. Flowchart. [file 12916_2021_2034_MOESM3_ESM.tif]
